# Case report: Twice-daily 15mA transcranial alternating current stimulation for adolescent major depressive disorder with suicidal ideation

**DOI:** 10.3389/fpsyt.2025.1669966

**Published:** 2025-09-23

**Authors:** Dan-dan Cheng, Yi-lin Yang, Zi-yi Yuan, Su Hong, Li Kuang

**Affiliations:** ^1^ Department of Psychiatric, The First Affiliated Hospital of Chongqing Medical University, Chongqing, China; ^2^ Mental Health Center, University-Town Hospital of Chongqing Medical University, Chongqing, China; ^3^ Psychiatric Center, The First Affiliated Hospital of Chongqing Medical University, Chongqing, China

**Keywords:** major depressive disorder, suicidal ideation, transcranial alternating current stimulation, adolescent, accelerated protocol, case series

## Abstract

**Background:**

Adolescent major depressive disorder with suicidal ideation (MDD-SI) poses significant treatment challenges and carries substantial mortality risk, while existing interventions often demonstrate limited acute efficacy for suicidal ideation (SI).

**Methods:**

This two-week prospective case series investigated the feasibility and preliminary therapeutic effects of high-frequency transcranial alternating current stimulation (tACS) administered twice daily. Seven adolescents with MDD-SI underwent 40-minute tACS sessions (77.5 Hz, 15 mA) twice-daily over a two weeks for a total of 20 sessions, in conjunction with stable pharmacotherapy.

**Results:**

The accelerated tACS protocol was well-tolerated with no adverse events. It demonstrated significant reductions in both depressive symptoms and SI within two weeks (all *P* < 0.05), predominantly in the first week. Treatment response varied based on psychiatric and medical comorbidities, indicating potential moderators.

**Conclusion:**

These preliminary findings suggest the potential of accelerated high-frequency tACS as a potential crisis intervention for adolescent MDD-SI, while highlighting the need for controlled clinical trials to establish efficacy and optimize stimulation parameters.

## Introduction

Major depressive disorder with suicidal ideation (MDD-SI) in adolescents represents a critical public health challenge, characterized by high prevalence rates (2.7%-45.1% in China) (1) and substantial mortality risk. Globally, suicide remains the second leading cause of adolescent mortality, typically progressing through a clinical trajectory from suicidal ideation (SI) to attempt, and completed suicide (2). The urgency is compounded by the fact that 30-60% of adolescents with MDD present psychiatric comorbidities including anxiety disorders, attention deficit hyperactivity disorder(ADHD), or metabolic conditions (e.g., obesity, diabetes), which collectively impair social functioning and elevate suicide risk (3–5). Notably, emerging evidence suggests that metabolic dysregulation and neuroinflammation (e.g., hypothalamic-pituitary-adrenal axis dysfunction) may establish a self-perpetuating “metabolic-emotional” cycle (4), rendering conventional pharmacotherapy and psychotherapy insufficient for acute suicidality.

Noninvasive brain stimulation (NIBS) has gained traction as a potential therapeutic alternative for adolescent MDD-SI. Although electroconvulsive therapy (ECT) demonstrates rapid anti-suicidal effects, its clincal utility is limited by generalized seizures introduction, cognitive side effects, and social stigma (6). Similarly, while accelerated theta-burst stimulation (TBS) protocol shows promise for acute symptom relief, their dependence on neuronavigation, associated risks (e.g., headache and hypomania), and lack of large-scale adolescent data restrict widespread adoption (7).

Transcranial alternating current stimulation (tACS) at 15mA/77.5Hz presents a distinct approach by directly modulating limbic structures (e.g., hippocampus, amygdala) critically involved in emotional processing, thereby aiming to restore neural synchrony. This parameter selection is based on computational modeling and clinical evidence suggesting its enhanced efficacy for deep limbic modulation (8, 9). Adolescents with MDD often exhibit dysfunctional prefrontal-limbic connectivity and disturbed neural synchrony within these circuits (10). Mechanistically, tACS may directly target these aberrant oscillatory activities and restore network balance, thereby alleviating depressive symptoms. Although direct evidence in adolescent populations remains limited, the neuromodulatory mechanisms of tACS align closely with core pathophysiological features of youth depression, providing a theoretical rationale for its application in this group.

Crucially, the developing adolescent brain exhibits heightened neuroplasticity within prefrontal and limbic circuits (11). Given evidence that adolescents may demonstrate increased responsiveness to NIBS techniques (12), it is plausible that this heightened neuroplasticity also enhances their sensitivity to the oscillatory entrainment effects of tACS. Evidence from adult trials supports its promising efficacy and favorable tolerability profile, both as a monotherapy and as an adjunct to pharmacotherapy (9, 13). Accelerated twice-daily protocols further suggest potential for rapid antidepressant effects in treatment-resistant depressions (14). However, existing tACS’s studies systematically excluded high-risk patients (HAMD suicide item ≥3), leaving a critical gap regarding its anti-suicidal potential.

This prospective case series represents a preliminary clinical investigation of twice-daily high-frequency tACS (15mA, 77.5Hz) in a small cohort of adolescents with MDD-SI. As an early-phase investigation, we focus on three translational advantages of this protocol: 1) the potential to modulate both cortical and limbic circuits through dual daily stimulation sessions, 2) apparently favorable tolerability critical for adolescent populations, and 3) operational simplicity allowing crisis intervention in general psychiatric settings. By systematically documenting feasibility, safety profiles, and preliminary response patterns in this high-risk cohort—including those with severe suicidality (HAMD suicide item ≥3) typically excluded from controlled trials—we seek to provide foundational data for future randomized clinical studies. The case series design permits granular observation of individual trajectories, which may inform patient selection and outcome measurement strategies for subsequent trials. Should this approach demonstrate acceptable safety and promising signals of efficacy, it might be further developed as a potential non-pharmacological intervention for adolescent psychiatric emergencies.

## Methods

### Participants

This two-week prospective case series recruited adolescents aged 12–17 years with MDD from the psychiatric outpatient and inpatient departments of Chongqing Medical University between January and March 2025.

Eligible participants met DSM-5 diagnostic criteria for MDD as confirmed by the Mini International Neuropsychiatric Interview for Children and Adolescents (MINI-Kid 5.0) and presented with clinically significant depression (HAMD-24 score ≥21) and active suicidal ideation (BSI-CV score ≥12 and HAMD-24 suicide item score ≥3). Written informed consent was obtained from all participants and their legal guardians.

We excluded individuals with severe somatic or neurological disorders (including epilepsy and traumatic brain injury), active substance abuse, skin lesions at electrode placement sites, implanted electronic devices (e.g., pacemakers), or prior exposure to neuromodulation therapies (ECT or rTMS). Additionally, participants were withdrawn from the study if they voluntarily requested discontinuation, showed clinical deterioration (worsening depression or increased suicide risk), developed new psychiatric comorbidities that affected outcome assessment, or experienced serious adverse events (e.g., seizures or severe skin irritation) or repeated non-adherence to the tACS protocol.

The study protocol was approved by the Institutional Review Board of Chongqing Medical University (Approval No. 2024-562-01) and conducted in accordance with the ethical principles of the Declaration of Helsinki and Good Clinical Practice guidelines.

### Permitted medications

All participants were required to maintain their existing psychotropic medication regimens at fixed doses for a minimum of four weeks prior to study enrollment, with no dosage adjustments permitted throughout the intervention period. This stability criterion was implemented to control for potential confounding effects of pharmacological changes on treatment outcomes, thereby allowing for more reliable attribution of any observed clinical effects to the tACS intervention.

### tACS intervention protocol

Given the absence of pediatric-specific tACS parameters, this case series study exploratorily applied a protocol (77.5 Hz, 15 mA) adapted from adult trials which have reported safety and preliminary efficacy for MDD. The primary aim was to assess the feasibility and tolerability of translating this paradigm to adolescents with MDD-SI, providing initial data for future pediatric-specific dose-finding studies.

The study utilized the Nexalin ADI device (Nexalin Technology, Inc., USA) to deliver twice-daily 15 mA/77.5 Hz tACS stimulation. Electrode placement followed the International 10–20 System for EEG electrode positioning, with three specialized electrodes (4.45 cm × 9.53 cm) positioned over the prefrontal cortex (targeting Fp1, Fpz, and Fp2 regions) and additional electrodes (3.18 cm × 3.81 cm) placed on the bilateral mastoid processes. Certified technicians administered the interventions twice daily (morning sessions between 08:00-11:00 and afternoon sessions between 14:30-17:30) from Monday through Friday. Each 40-minute treatment session was repeated over two consecutive weeks, totaling 20 sessions. Consistent with accelerated neuromodulation protocols, we maintained a minimum 4-hour interval between consecutive sessions to optimize treatment efficacy while minimizing potential neural adaptation effects.

### Clinical assessments

Baseline demographic information including sex, age, illness duration, age at onset, and body mass index (BMI) were systematically collected. Depressive symptom severity were assessed using both clinician-administered (24-item Hamilton Depression Rating Scale, HAMD-24) and self-report (Patient Health Questionnaire-9, PHQ-9) measures. Treatment response was operationalized as a ≥50% reduction in HAMD-24 scores from baseline, while remission was defined as a post-treatment HAMD-24 score ≤8. Suicidal ideation was specifically evaluated using the validated Chinese version of the Beck Scale for Suicide Ideation (BSI-CV).

All assessments were conducted at three time points: baseline (pre-intervention), week 1 (mid-intervention), and week 2 (post-intervention).

Adverse events (AEs) were monitored through a comprehensive protocol aligned with Common Terminology Criteria for Adverse Events v5.0 (CTCAE) (15). This included standardized recording of vital signs (blood pressure, heart rate) pre- and post-session, systematic documentation of neurological and psychiatric symptoms using tACS-adapted descriptors (9, 13), and clinician-administered severity grading.

### Safety monitoring protocol

For inpatient participants, we implemented a comprehensive safety management system in locked psychiatric wards. Two board-certified psychiatrists with at least associate chief physician qualifications independently conducted standardized suicide risk assessments using validated rating scales. Nursing staff performed structured safety rounds every two hours, supplemented by continuous electronic monitoring (CCTV surveillance and bed alarm systems) to ensure 24-hour safety oversight. All inpatient units maintained strict protocols for environmental safety, including removal of potential self-harm objects and controlled access to patient areas.

For patients receiving outpatient care, we implemented standardized safety measures. Primary caregivers were provided with structured education covering essential safety protocols, including proper medication custody procedures (such as locked storage) and requirements for continuous 24-hour patient supervision. To maintain close monitoring, the research team established daily contact through secure communication channels, utilizing encrypted messaging platforms and dedicated phone lines. Additionally, we implemented an emergency response protocol to guarantee that all outpatient participants could access immediate medical treatment within 15 minutes should urgent situations arise.

### Statistical analysis

All statistical analyses were conducted using SPSS version 25.0 (IBM Corp). We first assessed data distribution characteristics using the Shapiro-Wilk normality test. Continuous variables with normal distributions were summarized using mean ± standard deviation (SD), while non-normally distributed variables were reported as median with interquartile range (IQR). Given the expected non-normal distribution of psychiatric rating scale data in this small sample, we employed nonparametric tests for primary analyses. Within-group changes in clinical outcomes were analyzed using the Wilcoxon signed-rank test for paired samples. All tests were two-tailed, with statistical significance set at *P* < 0.05. For safety data, we conducted descriptive analyses of adverse event frequencies and severity ratings.

## Results

### Patient characteristics

**Figure 1:**
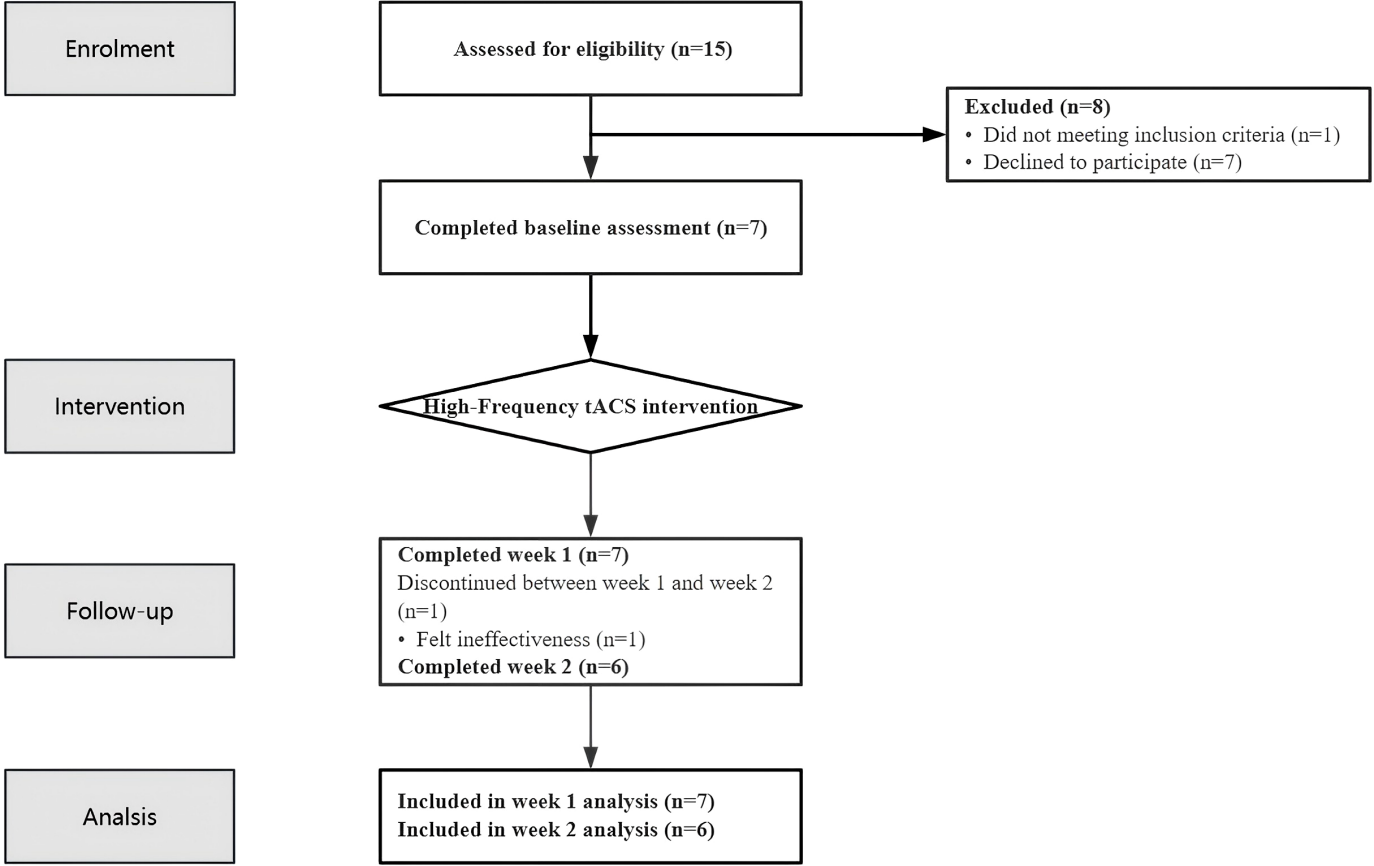
Flow chart.

A total of 15 potential participants were screened for eligibility in this study. Among them, seven patients declined to participate, and one was excluded for not meeting the inclusion criteria (see **Figure 1** Flow Chart). The final study cohort comprised seven MDD patients (2 males, 5 females; 4 inpatients, 3 outpatients) with a mean age of 13.67 ± 1.97 years (range: 12–17 years). The median duration of depressive symptoms was 10.05 months (IQR: 5.75-30.00 months) (1) (see **Table 1**).

**Table 1:** Clinical characteristics and outcomes of study participants.

Measure	Patient							Group
	1	2	3	4	5	6	7	
Sex	F	F	F	M	M	F	F	5F; 2M
Inpatient	No	No	Yes	No	Yes	No	Yes	
Age at Onset, years	13	15	11	11	14	11	13	12.50 (1.76)
Current depressive episode duration, months	6	48	12	24	9	5	6	10.05 (5.75, 30.00)
Age, years	13	17	12	13	15	12	14	13.67 (1.97)
BMI, kg/m^2^	18.36	33.09	17.85	19.82	20.72	16.44	23.12	21.05 (6.09)
Comorbidity	None	T2DM	None	ADHD	None	None	None	

Data presented as mean ± SD, median (IQR), or n (%) as appropriate; F, female; M, male.

Notably, two patients presented with comorbid conditions: one with ADHD and another with Type 2 Diabetes Mellitus (T2DM), stably managed with adjunctive methylphenidate and metformin respectively. All participants maintained stable psychotropic regimens: SSRIs (primarily sertraline) and adjunctive atypical antipsychotics (predominantly aripiprazole), with all psychotropic medications doses remaining stable for at least four weeks preceding study enrollment (see **Appendix 1** for regimens).

### Primary clinical outcomes

The study cohort comprised seven enrolled participants, with six patients (85.7%) completing the full treatment protocol. One patient (Case 7) discontinued the intervention after 10 sessions (Week 1) due to suboptimal therapeutic response (15.38% reduction in HAMD-24 scores) and subsequently transitioned to ECT.

**Figure 2:**
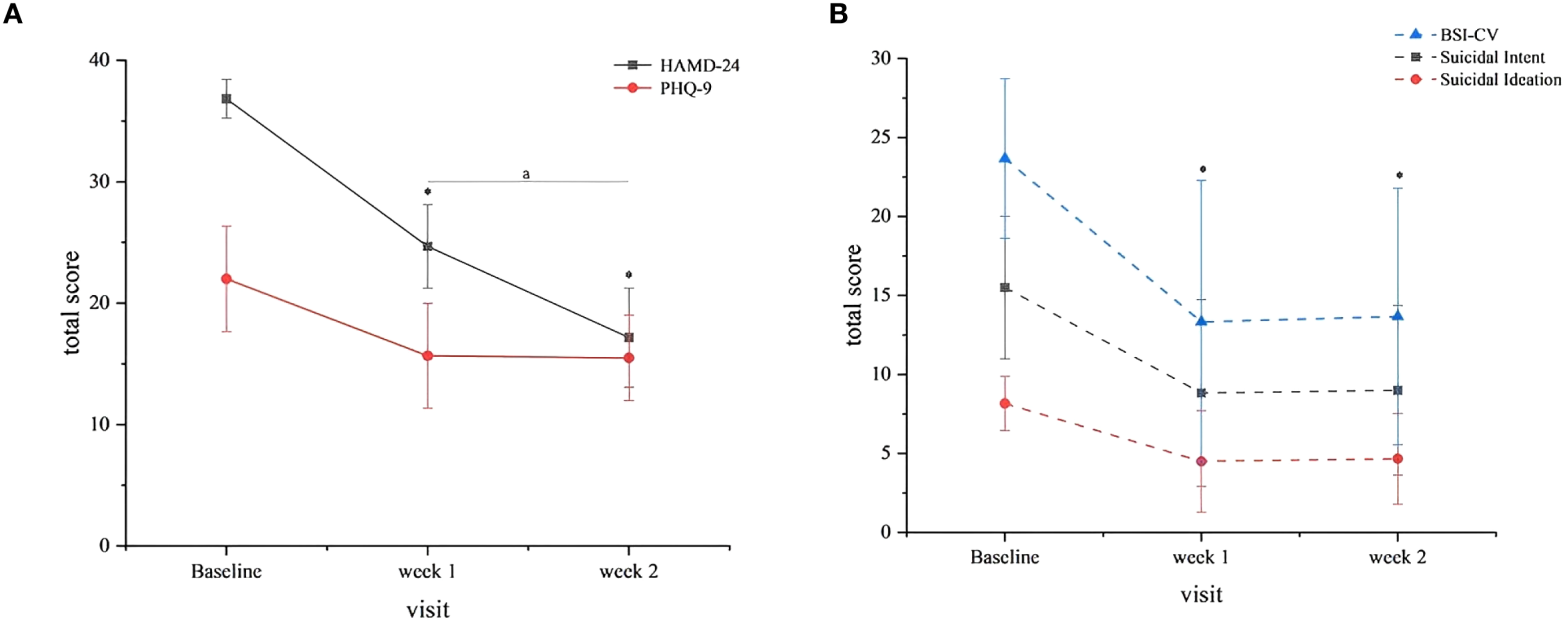
Clinical outcomes. **(A)** HAMD-24 and PHQ-9 Scores. Both scales decreased significantly from baseline to weeks 1 and 2 (*P*<0.05). Error bars represent standard deviation (SD). Symbol: (*) vs. baseline (*P*<0.05). **(A)** Week 2 vs. Week 1 (HAMD-24 only, *P*=0.042). **(B)** BSI-CV total and subscale scores suicidal ideation and intent subscales mirrored total score reductions (*P*<0.05). Error bars represent SD. Symbol: (*) vs. baseline (*P*<0.05).

The intervention demonstrated significant therapeutic efficacy across multiple symptom domains. Longitudinal analysis revealed statistically significant reductions in both depressive symptoms (HAMD-24 and PHQ-9) and suicidal ideation (BSI-CV) from baseline to weeks 1 and 2 (all *P* < 0.05; **Table 2**). By week 2, four of six treatment completers (66.7%) had achieved clinical response (≥50% reduction in HAMD-24 scores), though no participants met full remission criteria (HAMD-24 ≤8), suggesting persistent albeit reduced symptom burden. The temporal pattern of symptom improvement, as illustrated in **Figure 2** (n = 6 completers), showed the most substantial reductions occurring during the initial 10 treatment sessions (baseline to week 1), with more modest gains thereafter. Nonparametric comparison of week 2 versus week 1 outcomes demonstrated continued significant improvement in HAMD-24 scores (Z = -2.032, *P* = 0.042), while PHQ-9 and BSI-CV scores showed stabilization (both *P* > 0.05), indicating differential trajectories across symptom domains.

**Table 2:** Comparison of PHQ-9、HAMD-24 and BSI-CV scores pre-and post-treatment.

Outcomes	Patient							Group	Z	P
	1	2	3	4	5	6	7			
HAMD-24										
Baseline	38	37	35	35	39	37	39	37.14 (1.68)		
week 1	27	29	25	25	19^*^	23	33	25.86 (4.45)	-2.371	0.018^#^
week 2	22	21	14^*^	12^*^	19^*^	15^*^	/	17.17 (4.07)	-2.207	0.027^&^
PHQ-9										
Baseline	25	20	26	24	14	24	21	21.86 (3.98)		
week 1	20	10	16	21	12	15	21	16.43 (4.43)	-2.207	0.027^#^
week 2	21	18	14	15	14	11	/	15.50 (3.51)	-2.023	0.043^&^
BSI-CV										
Baseline	31	26	25	23	16	21	32	24.86 (5.58)		
week 1	24	19	15	17	1	4	29	15.57 (10.10)	-2.371	0.018^#^
week 2	22	22	13	12	13	0	/	13.67 (8.12)	-2.201	0.028^&^
Subscales of BSI-CV										
Suicidal Ideation										
Baseline	9	9	10	8	8	5	9	8.29 (1.60)		
week 1	8	6	6	6	1	0	7	4.86 (3.08)	-2.371	0.018^#^
week 2	8	7	5	3	5	0	/	4.67 (2.88)	-2.226	0.026^&^
Suicidal Intent										
Baseline	22	17	15	15	8	16	23	16.57 (5.00)		
week 1	16	13	9	11	0	4	22	10.71 (7.34)	-2.375	0.018^#^
week 2	14	15	8	9	8	0	/	9.00 (5.37)	-2.023	0.043^&^

^*^Response defined as HAMD-24 score ≥50% decrease from the baseline; ^#^Baseline-to-Week 1 analyses included all patients (n=7); ^&^Week 2 outcomes were analyzed only in completers (n=6).

### Longitudinal outcomes in comorbid cases

Extended six-week follow-up of patients with comorbid conditions revealed distinct clinical trajectories that warrant careful consideration. In Case 4 (ADHD comorbidity), the patient demonstrated remarkable clinical improvement, achieving full remission (HAMD-24 = 3) by the week 8 assessment (6 weeks post-treatment). This was accompanied by sustained reductions in both self-reported depression (PHQ-9) and suicidal ideation (BSI-CV) scores. Notably, caregiver reports documented meaningful behavioral changes including: 1) significantly improved social engagement and initiative, and 2) increased frequency and intensity of positive emotional expression.

Contrasting this positive outcome, Case 2 (T2DM comorbidity) experienced a serious adverse event at week 4 (2 weeks post-treatment), involving a non-fatal suicide attempt through antidepressant ingestion following intense family conflict. A comprehensive clinical evaluation identified multiple contributing risk factors: i) severely compromised family support systems, ii) paternal history of poorly controlled diabetes potentially modeling maladaptive health behaviors, iii) alarmingly low adherence to diabetes management protocols (diet/exercise compliance <30%), and iv) a strained therapeutic alliance that may have limited treatment efficacy. This case underscores the critical importance of implementing robust psychosocial support systems when treating adolescent MDD patients with complex medical comorbidities.

### Safety and tolerability profile

The intervention demonstrated an excellent safety profile throughout the study period. No severe adverse events (including commonly reported concerns such as headache or tinnitus) were observed in any participants. This favorable tolerability data supports the feasibility of implementing this treatment approach in adolescent populations, including those with comorbid psychiatric and medical conditions. The absence of significant adverse effects is particularly noteworthy given the complex clinical presentations in our study cohort.

## Discussion

### Efficacy outcomes

As an exploratory case series (n=7), this study provides preliminary evidence regarding the potential of an accelerated twice-daily high-frequency tACS protocol (77.5 Hz, 15 mA) for adolescents with acute MDD-SI. Several clinically relevant observations emerged: first, a response rate of 66.7% among treatment completers, with statistically significant reductions in both depressive symptoms (*P* < 0.05) and suicidality measures across all participants; second, sustained antidepressant effects suggested by continued HAMD-24 score reduction throughout treatment (*P* = 0.042); and third, a temporal response pattern wherein 60-70% of total symptom improvement occurred within the first 10 sessions, indicating a rapid early response trajectory. This front-loaded efficacy profile appears consistent with response patterns observed in adult tACS study (13). However, the open-label design, lacking a sham control, and concurrent stable pharmacotherapy preclude attribution of these improvements solely to tACS. Although the rapid reduction following tACS initiation suggests a specific neuromodulatory contribution, observed clinical effects likely reflect combined influences.

The marked discrepancy between response (66.7%) and remission (0%) rates underscores the substantial residual symptom burden in this severely ill cohort. The universal lack of remission likely reflects both the high baseline severity and sample limitations. This pattern aligns with observations from extended 4-week twice-daily tACS protocols in adults with severe depression (14), suggesting that accelerated tACS may primarily facilitate rapid crisis stabilization—a primary goal in acute settings—while indicating that most responders will require longer-term, multimodal strategies to achieve full remission and functional recovery.

### Comorbidities

Differential outcomes in comorbid cases offer preliminary insights into potential response moderators. The patient with ADHD (Case 4) achieved full remission (HAMD-24 = 3) at 6-week follow-up, with parallel improvements in core ADHD symptoms including attentional control and emotional regulation. This synergistic effect may be explained by tACS’s dual neuromodulatory actions: 1) enhanced of functional connectivity in dorsolateral prefrontal cortex (DLPFC) networks, which might benefit both executive dysfunction in ADHD (16, 17) and affective regulation in MDD; and 2) modulation of inflammatory markers such as interleukin-6, which has been hypothesized to influence impulse control (18). These observations suggest that tACS might be particularly suitable for MDD patients with comorbid executive dysfunction.

In contrast, the outcome in the T2DM-comorbid case (Case 2) highlights important considerations for patient selection and adjunctive care. Although initial symptom reduction was observed, the subsequent suicide attempt suggests a complex interplay between metabolic and psychiatric pathology: 1) severe obesity (BMI 33.09 kg/m²) and associated chronic inflammation may have compromised tACS efficacy through prefrontal metabolic dysfunction (18, 19); and 2) multiple risk factors, including history of non-suicidal self-injury, high stress reactivity, and poor diabetes management adherence, may have contributed to clinical decompensation that attenuated potential treatment benefits (20). This case underscores the necessity of comprehensive risk assessment and integrated psychosocial interventions when treating medically-complex adolescent with MDD.

### Safety and tolerability

The accelerated tACS protocol demonstrated favorable safety and tolerability in this adolescent MDD-SI cohort, with no severe adverse events reported. Mild and transient side effects were consistent with those reported in adult studies using identical parameters (9, 13, 14). The 15 mA intensity, though adapted from adult protocols, did not increase adverse reactions in adolescents, supporting its feasibility in this neurodevelopmental population. Moreover, tACS offers practical advantages over ECT—including no requirement for anesthesia, absence of seizure induction, and minimal risk of cognitive impairment—making it suitable for repeated or sustained application.

Adolescents’ heightened prefrontal-limbic neuroplasticity (10) may augment responsiveness to tACS. The 77.5 Hz stimulation targets limbic structures critically involved in emotional processing (e.g., amygdala, hippocampus), which are frequently dysregulated in depression. Rapid symptom reduction within the first week may reflect both the accelerated stimulation schedule and increased neuroplastic sensitivity in youth, suggesting a potential critical window for NIBS efficacy—though this requires further controlled investigation.

### Limitations and future directions

Several limitations must be acknowledged. The open-label design without sham control precludes definitive causal attribution of improvements to tACS, as placebo effects and natural disease fluctuation cannot be ruled out. Furthermore, concurrent pharmacotherapy limits isolation of tACS-specific effects. The use of adult-derived parameters, though tolerable, highlights the need for dose-finding studies optimized for the developing brain. Additional constraints include the small sample size, lack of biomarker data, and single-center recruitment, which limit statistical power and generalizability. Nonetheless, this study offers the first systematic evidence supporting the feasibility and safety of a twice-daily accelerated tACS protocol in this high-risk population. Future trials should prioritize multicenter, sham-controlled designs with larger samples, incorporate electrophysiological biomarkers (e.g., EEG), and include extended follow-up to confirm efficacy and establish adolescent-specific stimulation protocols.

## Data Availability

The original contributions presented in the study are included in the article/Supplementary Material. Further inquiries can be directed to the corresponding authors.
