# Supplementary material for: Case report: Twice-daily 15mA transcranial alternating current stimulation for adolescent major depressive disorder with suicidal ideation
Source: Front Psychiatry. 2025 Sep 23;16:1669966. doi: 10.3389/fpsyt.2025.1669966 (PMC12502979; doi:10.3389/fpsyt.2025.1669966)
Supplement: Supplementary file 1 [file DataSheet1.docx]

| Appendix 1. Medication Regimens of Study Participants | | | |
| --- | --- | --- | --- |
| Patient ID | Antidepressants, Dose (mg/day) | Antipsychotics, Dose (mg/day) | Other Medications, Dose (mg/day) |
| 1 | Sertraline, 100 mg QD | Aripiprazole, 5 mg QN | - |
| 2 | Sertraline, 100 mg QD | Aripiprazole, 5 mg QN | Metformin, 1 g BID |
| 3 | Sertraline, 100 mg QD | Quetiapine, 100 mg QN | - |
| 4 | Escitalopram, 15 mg QD | Aripiprazole, 7.5 mg QN | Methylphenidate, 18 mg QD |
| 5 | Fluvoxamine, 150 mg QD | Olanzapine, 2.5 mg QN | Eszopiclone, 3 mg QN |
| 6 | Sertraline, 100 mg QD | Aripiprazole, 5 mg QN | - |
| 7 | Sertraline, 100 mg QD | Aripiprazole, 5 mg QN | - |
